# Botanical Extracts for the Control of Plant-Parasitic Nematodes: Diversity, Modes of Action, Advanced Formulations, and Efficacy

**DOI:** 10.3390/plants15101502

**Published:** 2026-05-14

**Authors:** Juan Pablo Manjarrez-Quintero, Octavio Valdez-Baro, Heriberto Bayardo-Rosales, Juan Manuel Tovar-Pedraza, Alma Rosa Solano-Báez, Guillermo Márquez-Licona

**Affiliations:** 1 Laboratorio de Fitopatología, Subsede Culiacán, Centro de Investigación en Alimentación y Desarrollo, Culiacán 80110, Sinaloa, Mexico; jmanjarrez224@estudiantes.ciad.mx (J.P.M.-Q.); ovaldez222@estudiantes.ciad.mx (O.V.-B.); hbayardo225@estudiantes.ciad.mx (H.B.-R.); juan.tovar@ciad.mx (J.M.T.-P.); 2 Centro de Desarrollo de Productos Bióticos, Instituto Politécnico Nacional, Yautepec 62731, Morelos, Mexico; asolanob@ipn.mx

**Keywords:** *Meloidogyne*, botanical nematicides, plant-parasitic nematodes, essential oils, modes of action, oxidative stress, formulation strategies

## Abstract

Plant-parasitic nematodes (PPNs) cause substantial yield losses across a wide range of economically important crops worldwide, and the progressive withdrawal of synthetic nematicides due to toxicological and environmental concerns has created an urgent need for safer alternatives. Botanical extracts, owing to their chemically diverse secondary metabolites and multi-target nematicidal activity, represent one of the most thoroughly studied options. The present work synthesizes and critically evaluates the current state of knowledge on botanical extracts as nematicidal agents, encompassing phytochemical diversity, extraction methodology, nematicidal mechanisms, advanced formulation strategies, and the principal constraints limiting field-scale applicability. Research coverage has been markedly uneven: most studies have concentrated on a small set of plant families, particularly Lamiaceae, Asteraceae, Brassicaceae, and Meliaceae, with *Meloidogyne* spp. as the predominant target, while many other taxa remain underexplored. Proposed nematicidal mechanisms include oxidative stress, cholinergic interference, disrupted intracellular pH regulation, impaired detoxification, and induction of cell death; yet mechanistic integration through multi-omics approaches remains limited. Activity under laboratory conditions often declines markedly in soil, largely due to compound instability or volatility, a limitation that encapsulation and nanoemulsion formulations are beginning to address. Future research should prioritize standardized mechanistic studies and replicated field trials to bridge the gap between laboratory promise and practical nematode management.

## 1. Introduction

Quantifying crop losses caused by plant-parasitic nematodes (PPNs) is inherently difficult. These organisms inhabit the soil, and the symptoms they cause are indistinguishable from those produced by drought, nutrient deficiencies, or fungal infections: yellowing, stunting, and poor fruit set are commonly misattributed to abiotic stresses. Diagnosis is frequently delayed until after a full season of undetected population growth [1], contributing to the underestimation of published loss figures, which are already substantial. Plant-parasitic nematodes infect a broad spectrum of hosts and disrupt root architecture, impairing water and nutrient uptake. Furthermore, they predispose plants to secondary pathogen invasion, compounding the overall impact on crop production and complicating accurate loss assessment [2].

For several decades, synthetic nematicides were the standard response. Preplant fumigants were used to lower soil populations before sowing, while non-fumigant products were applied during planting and later in the season. In many applications, these products proved effective. The broader biological and environmental effects of these compounds, however, received considerably less attention at the time [3]. The toxicity profiles of many nematicides extend well beyond their intended targets, soil invertebrates, beneficial microorganisms, groundwater, and downstream aquatic environments [4]. Repeated use of structurally similar compounds has also created conditions favorable to tolerance development in nematode populations, a threat that is difficult to quantify in advance but straightforward in retrospect. Of equal or greater long-term concern, yet comparatively underexamined, is the chronic exposure of soil microbial communities to compounds whose effects on them were never the focus of the original risk assessments. Regulatory authorities in the European Union and other jurisdictions have responded accordingly: the list of registered nematicidal active ingredients has steadily shortened, with several widely used compounds banned outright or restricted to a fraction of their previous uses [5].

Against this backdrop, interest in plant-derived alternatives has grown substantially and is now widely recognized as a legitimate research direction. Secondary metabolite biosynthesis is a central component of plant defense strategy. The resulting chemical arsenal encompasses terpenoids, alkaloids, phenolics, sulfur-containing compounds, and numerous other structural classes, many of which include constituents with documented nematicidal properties [5]. In principle, working with a chemically diverse mixture rather than a single active ingredient confers an inherent resistance-delay advantage: a target organism must simultaneously overcome multiple independent disruptions rather than a single mechanism, substantially raising the evolutionary barrier to resistance. In practice, however, theory and field performance have diverged. Chemical composition shifts between harvests, the mechanistic picture is incomplete for most active compounds, and the decline in efficacy when transitioning from aqueous bioassays to field soil conditions has proven difficult to overcome in many cases. A rigorous appraisal of these limitations is indispensable for placing the available evidence in its proper context and for calibrating realistic expectations regarding field applicability.

## 2. Historical Overview of Plant Extracts in Nematode Management

### 2.1. Early Reports and Ethnobotanical Foundations

The concept that certain plant species could suppress soil-dwelling pests is longstanding [6]. Across different farming traditions, growers had long observed that incorporating certain plant materials into the soil frequently reduced pest pressure, including what is now recognized as nematode-mediated damage. These observations were eventually tested rigorously by researchers, and the initial results were promising. Studies conducted in the 1980s demonstrated that specific essential oil constituents (citral, geraniol, citronellol, and citronellal) had genuine nematicidal activity against plant-parasitic nematodes under in vitro conditions [1]. Notably, the potency of individual compounds varied in ways that tracked their chemical structure. This finding indicated that the observed bioactivity was not a vague or nonspecific phenomenon, but rather reflected specific interactions between defined molecular features and nematode biology, providing a biochemical rationale for practices previously justified only by tradition.

### 2.2. Emergence of Experimental Studies and Essential Oils

The 1990s saw this research mature [7]. Improved extraction methods and analytical chemistry tools enabled more precise characterization of essential oil compositions. As greenhouse experiments began to complement laboratory assays, some studies reported encouraging results: aromatic plant compounds and essential oils reduced nematode populations and root damage under controlled conditions, and, in some cases, their performance improved when combined with other inputs [8]. Essential oils consolidated their position as the dominant category within botanical nematicide research during this period. Concurrently, the taxonomic scope of studies widened. Essential oils from plants in the Asteraceae family, for example, were shown to interfere with egg hatching, juvenile survival, and reproduction across both in vitro and in planta conditions [9]. This kind of evidence helped solidify the field’s scientific credibility. It also rendered the limitations of the field more apparent: most studies were still confined to laboratory settings, the choice of target nematodes was taxonomically narrow, and virtually no work at the time was attempting to understand why these compounds worked, whereas mechanistic investigation was largely absent.

### 2.3. Transition Toward Mechanistic and Technological Approaches

**Figure 1:**
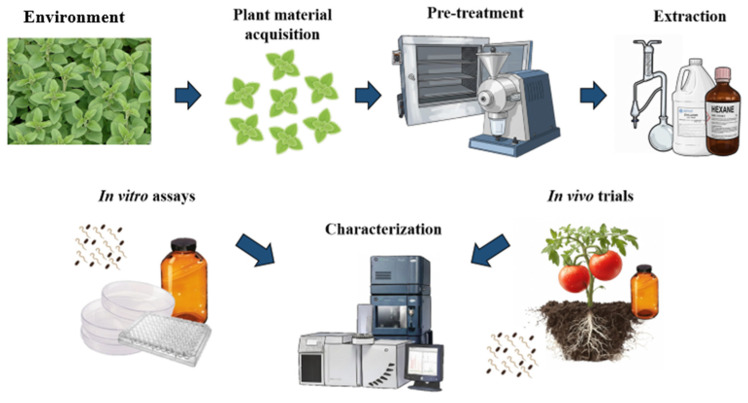
Integrated workflow for the development, characterization, and evaluation of botanical extracts against plant-parasitic nematodes.

Mechanistic questions have been present in the literature since at least the late 1980s, though they remained secondary to activity screening for several decades. Work on rishitin, a phytoalexin found in potato, showed early on that plant-derived compounds did not simply kill nematodes; they could repel them, hold them in a quiescent state, or kill them outright, depending on concentration and exposure duration [10]. These dose-dependent effects on behavior and physiology anticipated interest in sublethal exposures and behavioral manipulation that would develop more fully decades later. Recent review literature has noted an apparent paradox: the technical tools available to researchers (extraction methods, metabolite profiling platforms, and whole-organism bioassay systems) have never been better (Figure 1), yet work that actually tracks what happens inside a target nematode at the gene expression or cellular pathway level remains uncommon [11]. Biochemical assays and behavioral studies are more common in recent publications, and molecular tools have appeared in a small but growing number of studies [12]. Even so, the connection between phytochemical exposure and events within a nematode cell has been rigorously established for only a handful of compounds and species [12]. The field has matured considerably, but the gaps in mechanistic understanding are substantial and, as the rest of this review will show, they matter for translating laboratory activity into real-world efficacy [12].

In this context, the present review addresses a critical and underexplored question: what does the available evidence establish regarding the efficacy of botanical extracts as nematicidal agents, and where are the limitations? To address this, the review systematically examines: (i) phytochemical diversity and chemotaxonomic patterns underlying nematicidal activity; (ii) extraction techniques for volatile and non-volatile fractions and their influence on bioactive profiles; (iii) nematode target species and gaps in biological coverage; (iv) proposed mechanisms of nematicidal action, with emphasis on oxidative stress, neurotoxicity, ion homeostasis disruption, and detoxification inhibition; (v) formulation strategies aimed at improving field stability and efficacy; and (vi) the regulatory and commercialization landscape. Rather than cataloging positive results, this review provides a critical and integrative synthesis to identify the specific mechanistic, methodological, and translational gaps that must be addressed to advance botanical nematicides from laboratory promise to field utility.

## 3. Diversity of Plant Families and Extract Types: Chemotaxonomic Bias and Methodological Gaps

### 3.1. Phytochemical Distribution and Biosynthetic Specialization

Phytochemicals with nematicidal effects are concentrated in specific plant species, where certain families stand out for possessing secondary metabolites that control plant-parasitic nematodes. This chemotaxonomic distribution is the product of evolutionary processes through which certain plant lineages have developed and conserved biosynthetic pathways yielding secondary metabolites capable of acting as chemical defenses against organisms such as plant-parasitic nematodes. These changes have driven the development of specific, highly conserved biosynthetic pathways for producing secondary metabolites with nematicidal properties, such as the phenylpropanoid pathway, shikimic acid, malonate, and mevalonate [13].

A comprehensive review conducted by Mwamula et al. [13] demonstrated the nematicidal activity of botanical extracts from more than 348 plant species across 81 botanical families. The analysis revealed a concentration of species within specific families, with over 50% of the evaluated plant species belonging to the families Asteraceae, Fabaceae, Lamiaceae, Brassicaceae, Myrtaceae, Euphorbiaceae, and Apocynaceae. Within these families, Asteraceae, Fabaceae, Lamiaceae, Brassicaceae, and Myrtaceae represent approximately 30%, 20%, 17%, 10%, and 10% of the reported species, respectively. Furthermore, approximately 42% of the studies focused on a limited number of plant species, such as *Azadirachta indica*, *Melia azedarach*, *Eucalyptus* spp., *Ricinus communis*, *Satureja* spp., *Solanum* spp., *Allium* spp., *Thymus* spp., *Citrus* spp., and *Artemisia* spp., where *A. indica* represents approximately 16% of all reports, making it the most studied to date. These results demonstrate that, while the Meliaceae family is not among the families of nematicidal species with the greatest diversity, as mentioned above, a single species (*A. indica*) has received the most attention and study, and its extracts remain among the most studied botanical nematicides [13]. Each plant family specializes in a group of species with nematicidal effects, and these species concentrate specific secondary metabolites that function as nematicidal agents [13].

Within the Asteraceae family, research on marigolds (*Tagetes* spp.) is of great interest, as they produce a wide variety of phytochemicals with nematicidal effects. These compounds include sulfur compounds such as α-terthienyl and bitienyl [13], thiophenes (BBT, BBTOH, and BBTOAc) [14], flavonoids such as patuletin, quercetin, and patulitrin [15], and essential oils such as limonene, (Z)-β-ocimene, dihydrotagetone, (E)-tagetone, (Z)-tagetone, (Z)-ocimenone, and (E)-ocimenone [16].

Among the Fabaceae, research on *Crotalaria* spp. of particular interest focuses on molecules such as monocrotaline and other pyrrolizidine alkaloids, which have been identified as the main compounds responsible for nematicidal activity [17].

The Lamiaceae family is notable for a large number of aromatic species with nematicidal activity, including *Mentha*, *Origanum*, *Thymus*, *Rosmarinus*, *Ocimum*, and *Satureja*. In species of these genera, nematicidal activity is primarily associated with essential oils high in mono- and sesquiterpenes, such as carvacrol, thymol, carvone, p-cymene, eugenol, 1,8-cineole, and terpinene, among others [13].

Brassicaceae plant species, such as broccoli, cauliflower, cabbage, Brussels sprouts, mustard, turnip, and radish, exhibit nematicidal activity due to their high glucosinolate content, which, upon hydrolysis, produces biofumigant compounds such as allyl isothiocyanate. These compounds are chemically analogous to synthetic fumigants like methyl isothiocyanate. However, isothiocyanates derived from Brassicaceae are mostly considered less or minimally toxic, with the advantage of retaining effective nematicidal activity [13].

In the Myrtaceae family, four genera stand out for their nematicidal activity: *Eugenia*, *Eucalyptus*, *Melaleuca*, and *Syzygium*. In these genera, the nematicidal effects are primarily due to the composition of their essential oils. Unlike the Lamiaceae, whose composition is predominantly small herbaceous or shrubby plants, Myrtaceae species are generally large, woody plants, classified as trees. The composition of their essential oils is concentrated in molecules such as eucalyptol (1,8-cineole), eugenol, caryophyllene, terpinene, and pinene, among others, which are associated with nematicidal activity [13].

**Figure 2:**
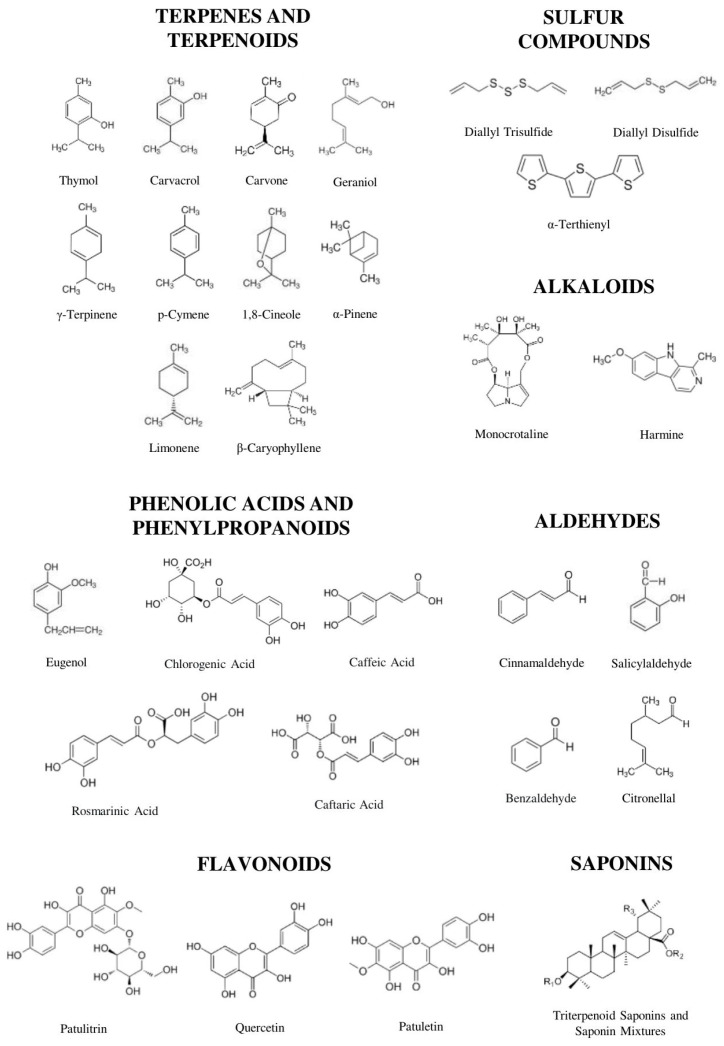
Representative chemical structures of major classes of botanical nematicidal compounds.

In summary, nematicidal activity reflects the specific biosynthetic specialization of the families, where plants concentrate different classes of secondary metabolites that defend against these plant pathogens. This chemotaxonomic pattern helps us directly predict extraction strategies, since maximizing extraction efficiency and bioactivity depends on the chemical composition of the target metabolites, highlighting the need for metabolite-directed extraction approaches in the development and formulation of botanical nematicides. Figure 2 summarizes representative phytochemical classes associated with nematicidal activity in botanical extracts reported in the literature, highlighting the chemical diversity underlying this bioactivity.

### 3.2. Extraction Techniques for Phytochemicals with Nematicidal Effects

Currently, research has focused primarily on three groups of extraction techniques for obtaining botanical extracts with nematicidal effects, which are broadly classified into volatile and non-volatile fractions. These three extraction techniques include the extraction of essential oils (volatile compounds) and the extraction using predominantly hydrophilic solvents (e.g., ethanol, methanol, water) or lipophilic to moderately polar solvents (e.g., dichloromethane, chloroform, hexane), both considered non-volatile [18,19].

The extraction method plays a fundamental role in the chemical profile and the nematicidal efficacy of botanical extracts, as different techniques selectively extract different classes of secondary metabolites. Consequently, the selection of the extraction method is crucial not only for the yield and composition of bioactive compounds, but also for their biological effectiveness, stability, and applicability in nematode management strategies [18,19].

#### 3.2.1. Essential Oils

Essential oils with nematicidal effects are mixtures of volatile lipophilic secondary metabolites, primarily mono- and sesquiterpenes, concentrated and extracted from aromatic plants using conventional or emerging (green) technologies. The extraction method is critically important because it directly affects oil yield, chemical composition, and nematicidal efficacy, as different techniques selectively yield distinct volatile metabolite profiles [20].

##### Conventional Extraction Methods

Conventional extraction methods include water-based techniques (hydrodistillation and steam distillation), organic solvent extraction (maceration and Soxhlet extraction), and cold mechanical pressing [18,20].

Hydrodistillation is based on the direct boiling of the plant material in water, while steam distillation is based on externally generated steam that passes through the biomass. In both methods, the water vapor entrains the volatile essential oil components, which are subsequently condensed and separated from the aqueous phase by density differences; residual water is then removed by decantation, yielding a nearly pure essential oil fraction. Although initially very similar (only differing in the contact of the plant material with boiling water or external steam), both methods produce essential oils with distinct chemical compositions, especially regarding heat-sensitive or highly volatile components [21,22,23].

Extraction methods using organic solvents employ nonpolar solvents (hexane, chloroform, petroleum ether, etc.) to solubilize the essential oils. However, these methods generally have low selectivity, as they also extract non-volatile lipophilic compounds such as waxes, pigments, and resins, thus reducing their purity [19]. Soxhlet extraction generally improves extraction efficiency by continuously recycling nonpolar solvents; however, it mostly yields oleoresins rather than pure essential oils, requiring further processing to purify and concentrate the essential oils [20].

Cold pressing preserves heat-labile compounds by avoiding thermal processing. However, it produces essential oils mixed with other lipophilic components and wastewater, as it is not selective and extracts all the liquid contained in plant cells [20,21]. In general, conventional methods are still widely used today; however, they have many limitations in selectivity, yield, purity, and chemical preservation, while offering clear advantages such as high scalability, low operating costs, and minimal or expensive specialized infrastructure.

##### Emerging and/or Ecological Extraction Methods

Emerging extraction technologies are divided into two methods: conventional assisted methods and advanced systems such as supercritical CO_2_ extraction [20,21,24].

Microwave-assisted extraction (MAE) is an improvement on hydrodistillation, which involves rapidly heating a mixture of water and plant material using microwaves, thereby reducing extraction time and energy consumption [20]. Ultrasound-assisted extraction (UAE) improves the release of essential oils by inducing acoustic cavitation using high-energy ultrasonic waves. This disrupts the cell wall, thereby increasing the efficiency and yield of the extraction process, as water readily penetrates the cell and enhances the entrainment of essential oils when heated to boiling [25].

Deep eutectic solvents (DESs) are primarily used as pretreatments prior to hydrodistillation because, like ultrasound, they promote cell wall disruption and improve subsequent essential oil recovery [26,27]. Supercritical CO_2_ extraction (SC-CO_2_) uses CO_2_ as a solvent in its supercritical state, combining the high solvency of a liquid with the low viscosity and high permeability of a gas. This process is modulated at moderate temperatures, allowing for high-yield, high-purity extraction of thermolabile compounds without organic solvent residues [20,21,28,29].

Overall, these emerging and/or green technologies improve extraction efficiency, chemical selectivity, and preservation of bioactive compounds compared to conventional methods. However, they are more complex to operate, require investment in appropriate infrastructure, and are more difficult to scale.

#### 3.2.2. Non-Volatile Botanical Extracts

Non-volatile botanical extracts comprise a chemically diverse group of phytochemicals, divided into hydrophilic and lipophilic extracts, both of which exhibit widely demonstrated nematicidal activity. Hydrophilic botanical extracts are mainly composed of phenolic compounds, alkaloids, saponins, glucosinolates, and glycosides, while lipophilic botanical extracts are mainly composed of carotenoids, sterols, alkaloids, isothiocyanates, sulfur compounds, etc. Similar to essential oils, the extraction technique significantly influences the yield, chemical composition of the metabolite profiles, and nematicidal efficacy [18,19].

##### Conventional Extraction Methods

Conventional extraction techniques include maceration, Soxhlet extraction, infusion, decoction, reflux, and percolation [19]. Maceration and Soxhlet extraction are identical to those used for essential oils. Infusion and decoction are extractions using boiling water, differing mainly in extraction time, while reflux maintains continuous contact between the solvent and the plant material through repeated evaporation-condensation cycles. Percolation relies on the continuous flow of solvent through compacted plant material to produce clarified extracts free of solids [19,30].

Conventional methods are the most widely used owing to their practicality, operational simplicity, and scalability. Their suitability, however, depends on the target metabolites and the plant material in question, as high-temperature conditions may degrade thermolabile compounds and reduce selectivity.

##### Emerging Extraction Technologies

Emerging methods for non-volatile compounds include UAE, MAE, pressurized liquid extraction (PLE), SC-CO_2_, pulsed electric fields (PEFs), enzyme-assisted extraction, fermentation, and DESs [19].

Methods assisted by UAE, MAE, and DESs improve upon conventional maceration techniques, as these technologies enhance cell permeability and metabolite release by disrupting the cell wall. SC-CO_2_ extraction can be targeted to moderately polar compounds by adding cosolvents such as ethanol or methanol [28]. PLE operates at elevated pressures and temperatures to improve solvent penetration and extraction efficiency, while PEF induces cell membrane poration without excessive heating [19,31]. Enzyme-assisted extraction and fermentation further promote metabolite release by enzymatically degrading the cell’s structural components. However, fermentation can chemically modify phytochemicals through secondary metabolism in microorganisms and, therefore, requires optimization to avoid degrading the metabolites of interest [19,32,33].

In summary, emerging methods improve extraction efficiency by increasing cell permeability and, in some cases, offer greater selectivity and less heat degradation. The main disadvantages are complexity and high operating and investment costs.

## 4. Nematode Targets

### 4.1. Root-Knot Nematodes (Meloidogyne *spp*.)

Species of the genus *Meloidogyne* are frequently cited in the literature as the primary model system for evaluating the efficacy of plant extracts, essential oils, and botanical formulations in both in vitro and host-plant bioassays [34,35,36,37]. Specifically, *M. incognita* and *M. javanica* are commonly used to assess juvenile mortality or immobilization, egg-hatching inhibition, and root-galling reduction, facilitating the accumulation of comparative data for this group. It is important to consider biologically relevant interspecific differences within this genus when interpreting results. For instance, *M. incognita* possesses a genomic architecture with duplicated and divergent regions linked to asexual reproduction, while *M. hapla* exhibits a distinct genomic pattern characterized by high recombination rates, which has significant implications for genetic variability and adaptive potential [38].

### 4.2. Root-Lesion Nematodes (Pratylenchus *spp*.)

Complementary to studies on *Meloidogyne*, evaluations have also been conducted on other agriculturally important plant-parasitic nematodes. In the case of lesion nematodes of the genus *Pratylenchus*, the literature includes species such as *P. brachyurus*, *P. penetrans*, *P. goodeyi*, and *P. coffeae*. These assessments are primarily carried out through in vitro bioassays to quantify immobilization or mortality and, to a lesser extent, through plant-based assays.

The evidence underscores that responses to treatment may vary substantially across nematode species and experimental contexts. In certain cases, specific treatments result in significant reductions in infection levels or nematode populations, whereas in others, their efficacy is limited. This variability appears to be influenced not only by the nematode species in question, but also by the particular attributes of the host plant [37,39,40,41,42,43].

### 4.3. Forest Nematodes (Bursaphelenchus xylophilus)

In parallel, a body of research has focused on nematodes of forest importance, particularly the pine wood nematode, *Bursaphelenchus xylophilus*. Within this context, essential oils, hydrodistillation fractions and waters, as well as crude extracts and purified metabolites, have been evaluated. These studies report direct nematicidal activity or associated physiological effects in nematodes [44,45,46,47,48,49].

### 4.4. Cyst Nematodes (Heterodera and Globodera)

Cyst nematodes of the genera *Heterodera* and *Globodera* have also been investigated in assays using plant extracts, organic amendments, and botanical formulations. These studies primarily focus on evaluating effects on egg hatching, viability, and/or population density under controlled and greenhouse conditions, thereby broadening the range of target taxa beyond root-knot nematodes [50,51,52,53,54].

### 4.5. Other Root Endoparasitic Nematodes

*Tylenchulus semipenetrans*, a citrus semi-endoparasite of broad economic relevance, has been the subject of both in vitro and greenhouse evaluations involving plant extracts and essential oils. These studies documented nematode paralysis or mortality, inhibition of egg hatching, and reductions in nematode populations in roots and soil. Notably, certain experiments also recorded improvements in host plant growth parameters, suggesting potential agronomic benefits beyond nematode suppression [55].

Similarly, *Nacobbus aberrans* has been included in investigations ranging from screening extracts of wild plant species to the chemical characterization of highly active fractions, with simultaneous evaluation of phytotoxicity. These findings emphasize that, although certain extracts may exhibit pronounced biological activity, they may also adversely affect germination or growth of host plants when applied at specific concentrations or with particular fractions [56,57,58].

### 4.6. Foliar and Tissue Nematodes

Regarding foliar and tissue nematodes, studies have been reported on *Aphelenchoides besseyi* and on species of the genus *Ditylenchus* (*D. destructor* and *D. dipsaci*). The available evidence mainly focuses on plant extracts and purified compounds that induce mortality or reduce nematode viability under controlled conditions, providing examples of botanical activity against nematodes with biological characteristics and ecological niches distinct from those of root endoparasites [59,60].

### 4.7. Research Gap and Bias Toward Meloidogyne

Despite the breadth of the reviewed literature, a clear research gap is evident, characterized by a disproportionate concentration of studies focused on the genus *Meloidogyne*, particularly *M. incognita* and *M. javanica*. As evidence of this bias, it has been estimated that approximately 80% of studies addressing the nematicidal potential of plant extracts focus exclusively on this genus [13].

This research trend restricts our ability to fully understand the nematicidal properties of botanical products, as it often sidelines other nematode groups that are also important from a phytosanitary perspective. The limited diversity of biological targets makes it difficult to generalize findings or draw broad conclusions about the efficacy of secondary metabolites, since different genera may respond quite differently to the same compound [61]. Furthermore, because of structural, physiological, and ecological differences, the modes of action of a single botanical extract are unlikely to produce identical biological responses in nematode groups with distinct survival strategies.

Consequently, it is imperative to diversify the biological models employed in botanical nematicide research. Overcoming the prevailing bias towards *Meloidogyne* would not only expand the spectrum of control to encompass species with contrasting biological traits, but also enable a more rigorous validation of the potential of secondary metabolites in increasingly complex and diverse agricultural systems.

## 5. Modes of Action of Botanical Extracts Against Plant-Parasitic Nematodes

**Figure 3:**
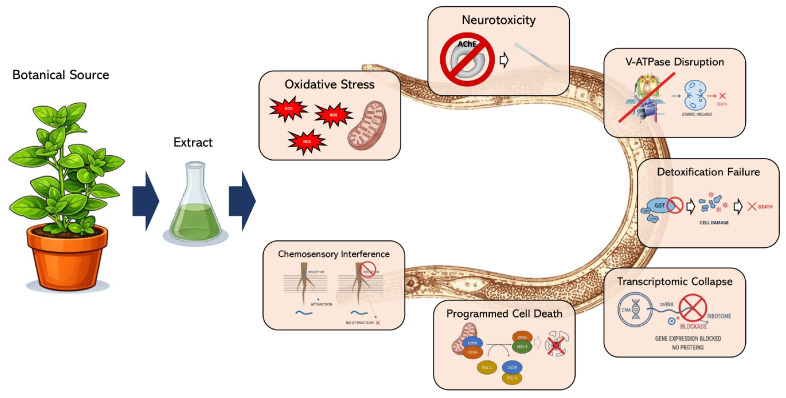
Multi-target mechanisms of action underlying the nematicidal activity of botanical extracts.

Botanical extracts do not kill nematodes as effectively as most synthetic nematicides do. Where conventional chemistry typically acts on a single, well-defined molecular target, an enzyme, an ion channel, a receptor, plant-derived compounds tend to hit nematode biology at multiple points simultaneously (Figure 3), with effects that compound rather than simply add up. Oxidative balance, neuromuscular signaling, intracellular proton gradients, detoxification capacity, protein and mRNA synthesis, and programmed cell death execution can be disrupted by the same compound or by different components of the same extract [62,63,64,65,66,67]. Studies integrating biochemical assays, high-resolution microscopy, transcriptomics, and multi-omics approaches have begun to shed light on these mechanisms at the molecular level, although the available evidence still comes mostly from a limited set of well-studied compounds and model systems [62,64,65]. Representative examples of the mechanistic categories identified in the literature are presented in Table 1. It should be noted that several of the mechanistic studies cited in this section employed *Caenorhabditis elegans* as a model organism. Although *C. elegans* is not a plant-parasitic nematode, it remains the only species in which stable genetic transformation, patch-clamp electrophysiology, and systematic loss-of-function analysis are routinely feasible, since plant-parasitic nematodes such as *Meloidogyne* spp. are obligate parasites that cannot be maintained in axenic culture under standard laboratory conditions. The molecular targets relevant to botanical nematicide action, namely acetylcholine receptors, GABA-gated chloride channels, V-ATPase, glutathione S-transferases, and the intrinsic apoptosis machinery, are functionally conserved between *C. elegans* and plant-parasitic nematodes, supporting the relevance of this model for hypothesis generation. *C. elegans*-derived evidence is presented here as mechanistic hypothesis-generating data; direct validation in plant-parasitic species is explicitly identified as a research priority.

### 5.1. Oxidative Stress and Redox Imbalance

Oxidative stress is the mechanism with the deepest and most consistent experimental support in this field. Broadly speaking, the process appears to involve an increase in ROS generation inside nematode cells beyond the capacity of their antioxidant defenses, followed by progressive molecular damage and, ultimately, cellular collapse [45,46,62,63,67].

This has been demonstrated across several nematode species and treatment types. In *Haemonchus contortus*, quercetin-induced mortality was tightly associated with the accumulation of reactive oxygen species (ROS) in neural tissue, specifically in the nerve ring and ventral nerve cord [75]. 5-aminolevulinic acid (ALA) treatment drove up lipid peroxidation and triggered antioxidant enzyme responses across four distinct nematode species *M. incognita*, *Heterodera glycines*, *Pratylenchus coffeae*, and *B. xylophilus* placing oxidative damage at the center of the toxic event rather than downstream of it [62,76]. Ultrastructural imaging confirmed the extent of the damage: widespread cellular disruption and autophagosome formation, consistent with systemic collapse rather than localized injury. Notably, acetylcholinesterase activity did not change after ALA treatment. This suggests that cholinergic interference was unlikely to drive the response and supports the idea that oxidative damage can act independently in this context [46,62].

Kalaiselvi et al. [63] further characterized the effect of *Artemisia nilagirica* essential oils on *M. incognita*, finding that nematode mortality closely tracked ROS accumulation levels. The genetic angle was then pursued using *C. elegans* mutants carrying defects in DNA damage checkpoints and apoptotic signaling: nematicidal activity was attenuated in these animals, which points to an intact ROS-dependent cell death pathway as a requirement for the lethal outcome not just a correlate of it [63,75].

The *C. elegans* model has proven particularly useful for gene-level analysis. When these animals were exposed to leaf extracts of *Solanum nigrum* and *Mentha arvensis*, gst-4 expression increased markedly. This gene encodes a glutathione S-transferase and is commonly used as a reporter of ROS-related cellular damage. Reverse transcription polymerase chain reaction (RT-PCR) data also picked up concurrent upregulation of hsp-16.2, hsp-4, hsp-6, and gpdh-1 genes associated with heat shock, endoplasmic reticulum stress, mitochondrial stress, and osmotic stress, respectively [77]. The simultaneous activation of several stress-response systems does not fit well with the idea of a single, narrowly targeted pathway; it more closely resembles a broad defensive response to pervasive cellular disruption.

*Citrus medica* essential oil reduced *C. elegans* survival under K_2_Cr_2_O_7_-induced oxidative stress, shortened lifespan, and impaired antioxidant capacity physiological changes consistent with redox disruption as an active contributor to nematicidal activity [78].

Collectively, these findings establish oxidative stress not merely as a correlate of nematicidal activity, but as an active and targetable mechanism. This has direct implications for the rational design of botanical nematicide formulations: compounds or mixtures that maximize ROS-generating capacity while minimizing antioxidant induction by the nematode represent a mechanistically informed selection criterion that remains underexploited in current screening workflows.

### 5.2. Neurotoxicity and Disruption of Neuromuscular Function

Rapid immobilization following exposure to botanical compounds is widely documented in the literature; however, the underlying mechanisms responsible for this response require careful mechanistic investigation. The most compelling mechanistic evidence links this immobilization to disruption of both cholinergic and inhibitory neurotransmission [66,68,79], and derives from direct electrophysiological recordings in *C. elegans* muscle preparations. Thymol, carvacrol, and eugenol all produced fast, concentration-dependent paralysis and blocked egg hatching.

Genetic dissection provided the next layer of evidence: animals lacking either the levamisole-sensitive acetylcholine receptor (L-AChR) or the UNC-49 GABA receptor were partially protected, which means both excitatory and inhibitory neuromuscular circuits are in play.

Patch-clamp recordings documented reduced acetylcholine and GABA evoked macroscopic currents; at the single channel level, channels opened less frequently but with normal conductance and open time when they did open. This pattern, characterized by reduced channel-opening frequency with otherwise unaltered conductance and open-time properties, is more consistent with allosteric modulation at a site distal to the agonist-binding pocket than with simple competitive blockade [80].

The relevance of these findings to actual plant-parasitic nematodes was addressed directly by Fanelli et al. [68]. Sublethal exposure of *M. incognita* to *Cinnamomum zeylanicum* and *Citrus aurantium* essential oils produced a two-phase pattern in Mi-ace-1 and Mi-ace-2, the genes encoding acetylcholinesterase: transcript levels first rose, then fell sharply. This sequential pattern of initial upregulation followed by transcriptional silencing was interpreted as neuronal stress progressing to cholinergic functional failure. This result was especially interesting because a similar transcriptional pattern had already been reported after oxamyl exposure, a synthetic nematicide with a well-characterized neurotoxic mechanism, suggesting the nematode nervous system is a primary target for certain botanical compounds, not an incidental casualty.

Gene expression data in *Meloidogyne luci* provide convergent mechanistic evidence from a complementary perspective: 1,4-naphthoquinone suppressed transcription of genes involved in GABAergic synapse function, meaning inhibitory motor control not just excitatory signaling is affected [64]. A separate line of work on trans-cinnamaldehyde in *C. elegans* yielded a convergent mechanistic profile through independent experiments: paralysis, partial protection in L-AChR and UNC-49 mutants, depressed acetylcholine- and GABA-evoked currents, and reduced channel-opening frequency; this convergent set of signatures is consistent with allosteric rather than competitive receptor interference [81].

### 5.3. Disruption of Proton Homeostasis and Osmoregulation

A third mode of action, mechanistically distinct from the first two, centers on inhibition of the vacuolar-type H^+^-ATPase (V-ATPase) an enzyme that manages intracellular pH, vesicle trafficking, nutrient uptake, and osmotic regulation. When this enzyme is inhibited, the resulting effects are both rapid and extensive [70,71,82]. The first clues came from phenotypic observations. Caboni et al. [70] noticed that aromatic aldehydes salicylaldehyde and cinnamaldehyde caused *M. incognita* to swell and lose turgor regulation. Scanning electron microscopy showed that changes in the cuticle surface were secondary to this internal imbalance, not its primary cause. The toxicity originated inside the cell.

Caboni et al. [71] then identified the molecular basis: α,β-unsaturated lactones, including tulipaline A, inhibit proton transport by covalently binding to conserved cysteine residues within the V-ATPase complex. Structure–activity relationship analyses established that this is a specific, targeted interaction dependent on the reactive electrophilic character of these compounds, not a nonspecific consequence of general chemical reactivity. Cheng et al. [82] later demonstrated the same mode of action for furfural acetone, an α,β-unsaturated carbonyl compound, suggesting that the same principle may apply to a wider group of reactive plant-derived molecules.

### 5.4. Inhibition of Detoxification Pathways

Nematodes are not passive targets. They have multi-phase xenobiotic detoxification systems, phase I (oxidative metabolism), phase II (conjugation), and phase III (efflux transport) that allow them to process and expel foreign compounds. When botanical compounds exceed the capacity of these systems or directly interfere with them, unprocessed toxic material can accumulate, feeding back into oxidative damage and cellular injury [64,65,83,84].

A rosin-based nematicide tested against *B. xylophilus* produced significant inhibition of glutathione S-transferase (GST) activity, together with reduced expression of the corresponding GST genes and increased levels of oxidative damage markers [65]. In *M. luci*, 1,4-naphthoquinone treatment caused repression of phase I and II detoxification genes, with concurrent upregulation of ABC transporters, suggesting that the nematode may have attempted to compensate through efflux once metabolic detoxification capacity was impaired, although that response was insufficient [64,83].

### 5.5. Global Transcriptomic and Metabolic Disruption

Not all documented botanical nematicidal effects are readily attributable to a single target-pathway framework. When *M. luci* was profiled transcriptomically after 1,4-naphthoquinone treatment, the main pattern that emerged was broad repression of genes encoding ribosomal proteins and spliceosome components, two functional categories so fundamental that their simultaneous disruption amounts to an attack on the cell’s basic molecular machinery, not a specific metabolic intervention [64]. Such broad transcriptional disruption is not readily explicable through any single mechanistic pathway.

Multi-omics profiling of *B. xylophilus* after exposure to a botanical nematicide revealed a similar pattern: lipid biosynthesis, carbohydrate metabolism, and apoptosis-related networks were all disrupted simultaneously [65,84]. Rather than exhibiting failure in a single pathway, the organism showed concurrent disruption across multiple functional networks.

### 5.6. Apoptosis and Programmed Cell Death

Oxidative stress, metabolic disruption, and genotoxic injury all share a potential downstream outcome: activation of the cell’s own death machinery. The genetic evidence for this was established by Kalaiselvi et al. [63], who showed that *C. elegans* mutants with loss-of-function defects in the apoptosis regulators cep-1/p53, egl-1, ced-3, and ced-4 were less sensitive to *A. nilagirica* essential oil than wild-type animals. The nematicidal activity depended on an intact intrinsic apoptosis pathway [63,75]. ALA treatment in separate experiments triggered autophagy-associated cell death, indicating that regulated cell death mechanisms other than classical apoptosis may also be involved [62].

### 5.7. In Silico Evidence Supporting Modes of Action

Molecular docking is common in recent botanical nematicide studies, often serving as a mechanistic explanation when direct biochemical evidence is not available. When used well, it has real value, but when docking output is presented as if it were experimental evidence, the distinction between a predicted interaction and a confirmed one tends to get lost, and that matters for the confidence with which any mechanistic conclusion can be drawn [65,66,85].

Acetylcholinesterase (AChE) is the most commonly predicted target, and in some cases, docking predictions are supported by experimental data. In the case of harmine quaternary ammonium derivatives evaluated against *B. xylophilus*, AChE inhibition was supported by both in vitro and in vivo evidence, and the structure–activity relationships inferred from docking were consistent with the experimental results [85]. This convergence of in vitro, in vivo, and in silico evidence constitutes the most robust mechanistic support available in this literature. In contrast, AChE inhibition predicted for imidazolylchalcones against *M. incognita* [86] or for fatty acids from cottonseed cake extracts [87] rests primarily on docking outputs combined with bioassay data, a weaker evidentiary base.

Other targets have been proposed. In *B. xylophilus*, piperine toxicity has been linked to glutamate-gated chloride channels (GluCl) through docking analyses and paralysis phenotypes resembling those produced by macrocyclic lactones [73]. There is also emerging interest in whether botanical compounds interfere with chemosensory signaling and host location: docking of *Zanthoxylum alatum* oil constituents and odorant-binding proteins have been invoked to account for the reduced infectivity of *M. incognita*, especially in nanoemulsified forms [74]. Glutathione S-transferases have also been proposed as possible detoxification-related targets in work on *Alpinia malaccensis* phytochemicals [88], consistent with transcriptomic and enzymatic evidence from *B. xylophilus* studies [65,83,84].

### 5.8. Synthesis and Knowledge Gaps

The mechanistic evidence, considered collectively, demonstrates genuine multi-pathway toxicity: botanical extracts can kill or impair nematodes through oxidative stress, neurotoxic interference, V-ATPase inhibition, detoxification suppression, widespread transcriptomic collapse, and programmed cell death activation, often through several of these pathways simultaneously. This mechanistic complexity likely underlies the potency of certain botanical compounds. It also renders rigorous mechanistic characterization substantially more challenging; for most compounds and target species, such characterization remains incomplete. Multi-omics studies in agronomically important *Meloidogyne* species are scarce. Docking predictions are frequently published without biochemical validation. The connection between what happens in a nematode cell after exposure to botanicals and how a product performs in the field has barely been explored.

## 6. Comparisons of In Vitro, In Vivo, and Field Studies

The botanical nematicide literature exhibits a structural imbalance that becomes immediately apparent upon systematic examination. The vast majority of published work evaluates nematode mortality, paralysis, or egg-hatching inhibition in laboratory assays under direct exposure conditions, artificial situations in which the compound of interest is in intimate contact with the target organism in a controlled, simplified medium. Strong activity under these conditions is frequently reported [13,34,35,36,59,67,89,90,91]. These assays serve legitimate screening purposes, but they share very little with the conditions under which the same compounds would need to perform in agricultural soil, a complex matrix of mineral particles, organic matter, microbial communities, water films, and competing chemical interactions [92,93,94].

Greenhouse and pot experiments have extended this work into more realistic settings, yielding encouraging results in some cases. Under controlled growing conditions, botanical products applied to tomato and other hosts infested with *Meloidogyne* have often produced noticeable reductions in gall formation, egg mass production, and nematode reproduction, particularly when substrates were sterilized or otherwise simplified [55,91,95,96,97,98]. Seed-derived extracts of *Crotalaria spectabilis* and finger millet have performed reasonably well even in unsterilized natural soils [17,99,100].

Where the picture becomes less reassuring is in the consistency of these results. Effects that appear promising at one evaluation point often weaken substantially in later assessments or disappear when substrate conditions are less favorable. García et al. [101] clearly showed that botanical extracts suppressed *M. incognita* in pitahaya at 30 and 60 days, but the effect was largely gone by day 90. This kind of temporal decay has been documented across the essential oil literature broadly and is attributed to volatilization, photodegradation, adsorption to soil particles, and microbial breakdown of the bioactive compounds [67,89,95,102,103,104]. The suppressive effect is therefore tied to the period during which the compounds remain active in soil. Once that persistence window ends, the level of suppression tends to decline.

Field trials open-soil conditions, full cropping cycles, and realistic variability are rare enough that individual studies stand out. Lu et al. [76] found that trans-2-hexenal suppressed *M. incognita* and improved tomato yield under field conditions at a level comparable to synthetic fumigants, and it remains one of the clearer examples of field-scale efficacy reported in this literature. Jang et al. [105] reported partial field suppression with a formulated *Waltheria indica* product, though the effect was not sustained throughout the season and was strongly dependent on formulation details and application strategy.

One of the most informative examples of the lab-to-field gap is the study by Eder et al. [106], who tracked a formulated garlic extract through multiple scales of evaluation. At successive scales, the study documented strong activity in vitro, moderate galling reduction in small pots, and then essentially no detectable effect in large-scale greenhouse trials with real agricultural soil. The authors’ conclusion, namely that laboratory-demonstrated nematicidal activity does not persist under realistic growing conditions, merits broader recognition than it has received [93,94,106].

Several strategies have been explored to address this translational gap. Applying phenolic acids as seed coatings partially suppressed *Heterodera glycines* in soybean, though results were variable across nematode races and no field data were collected [107]. Integrated programs combining botanical products with microbial biocontrol agents or low-risk synthetic nematicides have, in some greenhouse studies, brought reproduction factors below unity and achieved control comparable to commercial standards like fluopyram [108,109,110]. These results are encouraging; however, they derive from protected cultivation systems with manipulated substrates rather than from the variable, open conditions of commercial production agriculture.

A methodologically useful development in recent greenhouse work is the incorporation of ecological indicators alongside nematode population metrics. Zhao et al. [111] reported that *Asarum sieboldii* aqueous root extracts suppressed *M. incognita* while simultaneously supporting beneficial free-living nematode guilds and maintaining soil food-web structure, offering a broader assessment than nematode counts alone can provide [95,103,111]. Other approaches, such as silver nanoparticles mediated through plant systems, fumigants derived from cassava wastewater, have produced results approaching commercial fumigant performance in sealed or semi-closed substrate systems, but none have been tested at the field scale [112,113].

The pattern that emerges from this body of evidence is unambiguous: laboratory activity is abundant, greenhouse efficacy is conditional and often transient, and field evidence is rare. For the gap between these scales to close, research programs will need to treat soil persistence, formulation stability, realistic application rates, and long-term agronomic performance as primary evaluation criteria rather than as secondary considerations to be addressed once bioactivity has been established under simplified in vitro conditions [93,94,114].

### Comparative Efficacy of Botanical and Synthetic Nematicides

Evidence from comparative studies shows that botanical products can be highly active against nematodes under controlled conditions, although their performance relative to synthetic nematicides is not uniform. Under in vitro exposure, several plant-derived compounds display strong nematicidal activity. For instance, α-terpinene and α-pinene exhibited LC_50_ values of 36.22 and 47.49 mg/L, respectively, compared with 10.94 mg/L for oxamyl, indicating substantial but still lower toxicity [115]. In contrast, alkaloids isolated from *Triumfetta grandidens* showed effective concentration for 50% response (EC_50_) values of 0.09 µg/mL, slightly outperforming abamectin (0.13 µg/mL), which illustrates that certain purified phytochemicals can reach, or even exceed, the activity of synthetic standards [116]. At the same time, not all botanical compounds behave similarly. Rosmarinic acid, for example, was considerably less potent than copper sulfate (LC_50_ values of 0.95–1.18 vs. 0.073–0.154 mg/mL), pointing to a wide variability in efficacy among plant-derived molecules [117]. Interestingly, essential oils such as cinnamon oil have also been reported to outperform some synthetic compounds, with LC_50_ values far below those of fenitrothion, although these results come from specific systems and should be interpreted with care [118].

When moving from laboratory assays to greenhouse conditions, the picture becomes less favorable for botanical products. Several studies report that synthetic nematicides still provide more consistent suppression of nematode populations. For example, abamectin achieved up to 96.8% juvenile mortality and markedly reduced soil populations, whereas botanical extracts from *Citrullus colocynthis*, *Moringa oleifera*, and *Tagetes erecta* showed lower reductions under the same conditions [108]. A similar pattern was observed with algal extracts, which reduced nematode parameters by around 75% but remained below the ~99% suppression achieved by oxamyl [119]. That said, these botanical treatments were not inactive; in fact, they also enhanced plant defense responses, suggesting that their effects are not limited to direct toxicity. Taken together, these results point to a recurring issue: compounds that perform well in direct-contact assays often lose part of their efficacy once environmental factors such as degradation, soil binding, or limited mobility come into play.

Notwithstanding these limitations, there are documented situations in which botanical products perform on par with synthetic nematicides. In greenhouse pot experiments, integrated treatments combining botanical extracts with bioagents resulted in reproduction factors very close to those obtained with fluopyram [109]. The reproduction factor (RF), calculated as the quotient of final (Pf) to initial (Pi) nematode population density, is a standard agronomic metric; values below unity (RF < 1) indicate population suppression to below the established damage threshold. Likewise, azadirachtin reduced nematode populations in lettuce to a similar extent as fluopyram, although this effect did not persist in longer-cycle tomato crops [110]. This difference between short- and long-cycle systems is particularly relevant, as it suggests that persistence rather than intrinsic toxicity may be the limiting factor. In other words, botanical products may be sufficiently active, but their window of effectiveness is often shorter.

Recent work also shows that formulation plays a decisive role. A nanoformulated azadirachtin product achieved levels of nematode suppression comparable to, or even better than, fluopyram under field conditions, reducing final populations more effectively [120]. Similarly, studies that combine botanicals with organic amendments or beneficial microorganisms report improved outcomes compared to standalone applications [121]. These approaches do not rely solely on toxicity, but rather on combining multiple mechanisms, including direct nematicidal effects, improved plant vigor, and changes in the soil microbiome.

Overall, the evidence does not support a simple “botanical vs. synthetic” dichotomy. Instead, botanical nematicides appear to fall along a spectrum: crude extracts tend to be less effective under field-relevant conditions, whereas purified compounds, optimized formulations, or integrated strategies can narrow the gap and, in some cases, match or even exceed synthetic treatments. This variability highlights the importance of evaluating botanical products within realistic production systems, where factors such as formulation, application timing, and crop duration ultimately determine their practical value. These findings align with the conclusions of a recent systematic review confirming that performance variability is intrinsic to botanical nematicides and depends critically on formulation and application strategy [122].

To better illustrate these patterns, representative studies comparing botanical and synthetic nematicides across different experimental scales are summarized in Table 2.

**Figure 4:**
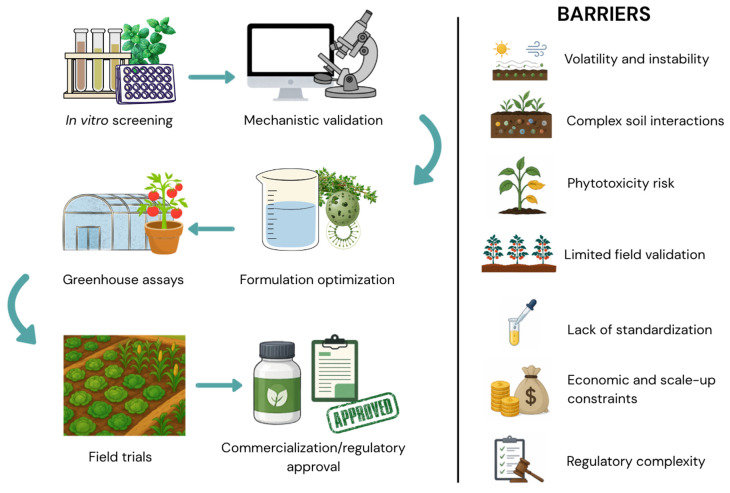
Development pipeline and major barriers limiting the field translation of botanical nematicides.

Despite the promising nematicidal activity reported under controlled conditions, the translation of botanical products into effective field applications remains limited. This discrepancy reflects multiple biological, chemical, and practical constraints that affect performance across experimental scales. The main stages involved in the development of botanical nematicides, along with the key barriers associated with each step, are summarized in Figure 4.

As illustrated in Figure 4, several factors contribute to the gap between laboratory efficacy and field performance. Volatility, chemical instability, and interactions with soil components can reduce the persistence and bioavailability of active compounds. In addition, risks of phytotoxicity, lack of standardization in extract composition, and limited field validation further constrain their practical application. Economic and regulatory challenges also play a critical role, particularly during scale-up and commercialization stages.

## 7. Advanced Formulation Technologies and Stability Enhancement

### 7.1. Physicochemical Restrictions of Crude Extracts for Agricultural Use

Currently, there is considerable scientific evidence at the laboratory level confirming that botanical extracts (essential oils and non-volatile extracts) possess nematicidal activity. However, their implementation as a management strategy in agriculture remains very limited due to physicochemical constraints and insufficient optimization of their formulations. Essential oils are highly volatile and have low chemical stability. Therefore, when applied under intensive agricultural production conditions, they tend to degrade rapidly due to various environmental factors, such as exposure to light and/or radiation, oxygen, and high temperatures. Even if they do not degrade, they tend not to leave residual effects in plant tissue, as many are not systemic and run off easily, evaporating rapidly. These limitations explain why there is a significant difference between the high nematicidal activity observed in in vitro studies and the limited effect, or the high quantities required for the same effect, in the field [93,95,114,123,124].

In addition, the low or nonexistent water solubility of terpenes (essential oils and non-volatile terpenes) and other lipophilic metabolites (lipids, chlorophyll, sulfur compounds, low-polarity phenolic compounds, etc.) reduces their availability in the soil, consequently limiting contact and residence time with nematode eggs and juveniles. Furthermore, it is important to consider that these limitations are exacerbated by poor adsorption onto soil particles (which varies with soil type) and by metabolic changes induced by the microbiota. This increases the difficulty for these extracts, with limited formulation strategies, to fully realize their potential in nematode control [102,103,104,114,123].

### 7.2. Agro-Environmental Factors Impacting Efficacy

Aside from the structure and physicochemical properties of botanical extracts, their nematicidal effectiveness is largely determined by agronomic and environmental conditions. The technical criteria for field application (concentration, dose, frequency, and residence time), storage parameters (temperature, humidity, darkness, container, volumetric oxygen, etc.), elements associated with crop development and management (phenological stage, plant physiology, stress status, etc.), and soil characteristics (texture type, pH, moisture, type and quantity of microbiota, type and availability of nutrients, amount of organic matter, etc.) all contribute to biological effectiveness in the field [92,93,94,114].

### 7.3. Uncontrolled Release Associated with Phytotoxicity Risks

One of the most significant limitations of crude botanical extracts without an optimized formulation is the uncontrolled release of nematicidal bioactive compounds. Rapid, uncontrolled dispersion could result in a rapid but uneven nematicidal effect (since those that survive have the opportunity to parasitize, since the extract is not released at a constant concentration). This necessitates high doses, which can cause phytotoxicity in crops (the severity of which varies by crop). For example, drench applications of essential oils with high concentrations of thymol and carvacrol have been linked to phytotoxic damage to the root system (deficient root development). This highlights the need to develop delivery systems that control release rate, thereby reducing doses, concentrations, and costs compared with extracts without proper formulation [93,124,125,126].

### 7.4. Use of Encapsulation and Nanoemulsions as Formulation Systems

To mitigate the limitations discussed, microencapsulation and nanoemulsion formulations are being used as potentially more effective approaches to improve key parameters such as stability and bioavailability. These technologies and methodologies modulate the release rate, shifting from first-order to near-zero-order release kinetics (from rapid, concentration-dependent release to constant release over time). These strategies can be relevant because the average life cycle of a plant-parasitic nematode is approximately 21–30 days. Therefore, a system that maintains constant release during this time could protect the crop from reinfestation by surviving juveniles or new offspring hatched from eggs already present before application [95,114,123,127].

Reducing the particle size to the nanoscale (<200 nm) increases the contact surface area, thus improving interactions with the nematode cuticle and maximizing biological efficacy [128,129,130].

### 7.5. Methods, Materials, and Optimization Strategies for Encapsulation

Currently, many encapsulation techniques have been employed for extracts with nematicidal effects, including nanoemulsification, spray drying, freeze-drying, extrusion-molding, fluidized bed coating, coacervation, molecular inclusion, and ionic gelation. Among these encapsulation techniques, nanoemulsification has achieved the widest adoption in this field owing to its operational simplicity, favorable scalability, and demonstrated biological effectiveness [131,132,133].

Encapsulation involves binding botanical extract molecules within materials that completely encapsulate them, thereby determining the formulation’s properties. These materials are diverse and chemically variable, but all serve the same purpose. Among them, natural polymers (alginate, chitosan, cellulose derivatives, starches, gums, pectins, etc.), proteins, saponins, and synthetic polymers stand out [131,132,133,134]. In addition to the type of encapsulating material, some important variables to consider in the formulation are the chemical composition of the extract, the extract-to-encapsulating material ratio, pH, processing temperature, mixing intensity, and ultrasound parameters [129,134]. To understand important aspects of encapsulation, physicochemical characterization is crucial, typically evaluating particle size, zeta potential, and polydispersity index, as well as evaluating their encapsulation efficiency, loading capacity, release kinetics, and stability under different conditions to which these formulations may be subjected, such as temperature variations, centrifugation, freeze–thaw cycles, exposure to UV radiation, oxygen, and storage time [129,130,135].

### 7.6. Biological Evaluation, Persistence, and Environmental Safety

To demonstrate high biological effectiveness and obtain regulatory approval for botanical extract-based products, final formulations must be evaluated in active and controlled crop fields. This evaluation assesses characteristics such as persistence in the soil, biotransformation by microbiota, and microbiological viability throughout the entire crop cycle and after the general application schedule [123,127,136]. This means that the effectiveness of bionematicides must be evaluated not only against plant-parasitic nematodes but also against the effects on non-target microorganisms, such as beneficial soil microorganisms, nematodes, and other free-living organisms, and host crops [94,103]. To be approved and safe to implement the use of botanical nematicides, it is necessary to consider a staged evaluation scheme, first encompassing in vitro laboratory tests, then in vivo tests in greenhouses or shade houses, and finally field tests, in order to create effective application strategies without damage to the environment [93,114,127].

## 8. Synergistic, Antagonistic, and Multitarget Interactions

### 8.1. Multicomponent Nature of Botanical Nematicides

Botanical extracts and essential oils are not isolated or purified molecules, but rather form part of a system with multiple compounds. These compounds include nematicidal substances, as well as inert and even antagonistic molecules. Therefore, the interactions between their components significantly determine their biological effectiveness. Interactions that generate a synergistic effect can increase efficacy by reducing doses and concentrations. Conversely, an antagonistic effect reduces biological effectiveness, either partially or completely. Considering these molecular interactions are highly relevant to developing high-performance formulations in the field, they are not commonly studied in research on the management of plant-parasitic nematodes with botanical extracts, leaving this line of research an area of opportunity [137].

Previously, the central idea behind the use of nematicides was that a purified compound would target only one specific goal. This was believed to be more specific and efficient. However, this is currently being replaced by a multi-site action hypothesis, which suggests that the phytochemical complexity of extracts confers greater effectiveness than isolated molecules. This advantage stems from both the synergistic effects of these phytochemical mixtures and the possibility that the nematode will not develop resistance. Looking in detail at how these mixtures of bioactive compounds act, many of them first disrupt or alter the nematode cell membrane, allowing the other compounds greater fluidity into the cell, where they target multiple cellular sites (Section 5). This coordinated bioactivity increases metabolic collapse and consequently the death of the nematodes [13,66,79,137,138].

### 8.2. Synergistic and Antagonistic Effects Among Different Essential Oils

Currently, synergy between essential oils against *B. xylophilus* has been demonstrated [138]. In this case, it was determined that mixtures of essential oils from *Cymbopogon citratus*, *Eucalyptus globulus*, *Foeniculum vulgare*, *Mentha piperita*, *Origanum vulgare*, *Rosmarinus officinalis*, *Salvia officinalis*, and *Satureja montana* had synergistic effects in almost all the mixtures evaluated. Among the most promising combinations, the 1:1 mixture of *M. piperita* and *R. officinalis* was of particular interest, as their essential oils individually showed nematicidal effects of 1.2% and 14.2% at 1 µL/mL, respectively. The combination was the most effective at 51.8%, demonstrating a high synergistic effect, with a 3- to 43-fold variation depending on the individual oil compared.

Most research on essential oil combinations focuses on mixtures of two types of essential oils; however, evidence indicates that mixtures of three essential oils can be more effective, outperforming both binary mixtures and individual essential oils. For example, the 1:1:1 mixture of essential oils of *Piper longum*, *Vitex negundo*, and *Vitex agnus*-castus showed superior effectiveness against *M. incognita*. This mixture increased J2 inhibition by approximately 10% compared to binary mixtures and by 10-20% compared to individual essential oils. Regarding egg hatching inhibition, the effect was 20-30% compared to individual essential oils and approximately 10% compared to binary mixtures, indicating that the synergistic effect is largely dependent on the nematode’s developmental stage [139].

### 8.3. Effect of Interactions Between Purified Compounds Derived from Essential Oils

The evaluation of interactions between purified compounds derived from essential oils is of particular interest, since many essential oil compounds are more active in their purified form. However, when mixed with other compounds from other essential oils, synergistic or antagonistic effects can occur. Ntalli et al. [66] demonstrated evidence of both synergistic and antagonistic effects against *M. incognita*. In this study, nine terpenes derived from essential oils were evaluated in two-component mixtures. Trans-anethole exhibited the highest frequency of synergistic interactions with most of the terpenes, while L-carvone showed the greatest antagonistic effect. The most effective synergy was observed in the geraniol/trans-anethole mixture, which was 12 times more effective than the individual compounds. Other mixtures with notable synergistic effects were trans-anethole/eugenol, carvacrol/eugenol, geraniol/carvacrol, and carvacrol/thymol.

It is important to note that the synergistic effect is not only determined by the type of compounds but also depends on their concentration and molar ratio. In some cases, the same pair of compounds can shift from synergistic to additive or even antagonistic effects depending on their proportions. In specific cases, synergy is achieved with a small addition of a single compound, resulting in effective interactions at mass ratios as low as 15:1 (1:0.066) [79].

Furthermore, the final concentration of the mixture is another crucial factor. Even with equal molar ratios (e.g., 15:1 of 1,8-cineole/caryophyllene oxide), mixtures evaluated at different total concentrations (0.048, 0.024, and 0.01 mg/mL) shifted from a synergistic effect to an additive effect at lower doses. Therefore, this must be carefully considered when formulating mixtures to achieve a synergistic effect [79].

### 8.4. Synergy Among Botanical Extracts

The synergistic or antagonistic effects among non-volatile, whole botanical extracts with nematicidal activity have not yet been extensively researched, representing a significant area of opportunity. To date, almost all research has focused on interactions between purified compounds or between essential oils, with little evidence on the effects of extract combinations or comparisons between crude extracts and their purified compounds. Furthermore, there is a significant lack of greenhouse or in vivo studies demonstrating synergy, as most research has been limited to in vitro tests.

While synergistic effects are often relevant in vitro, in vivo synergy presents a more complex picture due to several factors, such as conditions that closely resemble real-world scenarios and the evaluation of multiple factors, including effects on nematodes, crop physiological parameters, and so on. An example is the study published by Khairy et al. [140], who evaluated the nematicidal effectiveness of aqueous extracts of moringa leaves, canola leaves, stems, and roots, as well as neem leaves, against *M. incognita* under greenhouse conditions. The nematicidal effectiveness of both individual extracts and mixtures of two and three extracts was assessed by measuring the number of females, J2 larvae, eggs, reproduction factor, root gall index, and egg mass index. In addition, plant growth parameters (root and stem length, and fresh weight), leaf nutritional composition, and chlorophyll content were evaluated. However, there is still considerable discrepancy in the effects of the combinations: the ternary combination is better for some parameters, the binary combinations for others, and the individual extracts for others. This highlights the need for more complex mixtures, with varying molar ratios and concentrations, to better exploit the synergistic effects of complex extracts.

As with purified essential oil compounds, little evidence has been reported of interactions between purified compounds of non-volatile botanical extracts. One study demonstrates that purified chalcones exhibit high synergistic activity against *M. incognita* in mixtures of two or three chalcones, with the three-chalcone mixture showing the greatest effectiveness, achieving 99.1% mortality at only 4 µM. However, this high synergy is directly dependent on concentration, as the same mixtures of purified compounds, but at different concentrations, went from highly synergistic to moderately synergistic interactions [141].

### 8.5. Cross-System Synergies and Methodological Considerations

The more complex a mixture, the more effective multi-site nematicidal mechanisms can be. Therefore, an additional and complex form of synergy should involve mixtures of essential oils, non-volatile botanical extracts, and purified compounds. For example, the synergistic and additive effects of mixing lavender essential oil, aqueous lavender extract, and trans-anethole against *M. incognita* were determined. In these systems, with three very different types of components, it is important to consider the physicochemical properties and chemical composition in detail, since the observed interactions depend on multiple factors, such as the evaluation method (immersion versus fumigant activity), exposure time, and the stability of the compound. Volatilization, degradation, or destabilization over time could transform the interaction from synergistic to additive. Variability is also observed among *Lavandula* species (with different chemical compositions and physicochemical properties) [142].

### 8.6. Perspectives for Mixture Design

The body of research on multicomponent interactions underscores the need to employ improved statistical designs, such as mixture designs, to identify optimal mixtures of concentrations, molar ratios, compounds, or complete extracts. Variables to consider for this objective include the target nematode species, the developmental stage (larvicidal or ovicidal activity), the evaluation method (immersion or fumigation), the experimental scale (in vitro or in vivo), and the nematicidal mechanisms of action, etc. This is why the systematic (methodical, organized, and structured) analysis of the interactions between non-volatile botanical extracts, essential oils, oil/extract, as well as between purified compounds, represents one of the strategies with a high area of promising opportunity to achieve botanical control of nematodes with high efficiency, with low doses and with formulation and design suitable for its application in the field.

## 9. Ecotoxicological, Phytotoxic, and Non-Target Effects

### 9.1. Overview of Ecotoxicological and Non-Target Effects

Plant extracts and essential oils are frequently presented as candidate replacements for synthetic nematicides, primarily due to their natural origin and rapid degradation in the environment. However, the scientific literature indicates that bioactivity against plant parasitic nematodes does not necessarily equate to high ecological selectivity. Both experimental studies and comprehensive reviews concur that the compounds responsible for nematicidal activity may also impact non-target organisms and essential soil functions [94,104,113,143].

Ecotoxicological studies have shown that essential oils and botanical extracts may cause lethal or sublethal effects on soil invertebrates, microorganisms, and beneficial arthropods. These impacts often occur at concentrations similar to those used in nematode control experiments [94,104,113,144]. This limited selectivity arises because plant secondary metabolites act on fundamental physiological targets such as membrane integrity, redox balance, and nervous system signaling, thereby increasing the risk of unintended effects [94,143,145].

Further research suggests that when evaluating botanical nematicides, it is important to consider not only the plant compound itself but also its formulation and application. Comparative studies show that the same plant material can have markedly different ecological impacts depending on whether it is used as an essential oil, an aqueous extract, or a soil amendment. These differences can affect non-target nematodes and soil microbiota in unique ways [94,95,103]. Therefore, it is important to factor in non-target effects when developing and assessing plant-based nematicidal strategies.

### 9.2. Phytotoxic Effects on Host Plants

Phytotoxicity is a frequently reported non-target effect when using essential oils with nematicidal properties. Both classic and recent studies have demonstrated that oils like clove, oregano, and rosemary may inhibit seed germination, limit root growth, and cause visible seedling damage, especially in sensitive crops such as tomato [94,146,147]. Notably, the concentrations effective against nematodes often overlap with those that cause phytotoxicity, leaving a narrow margin between controlling nematodes and ensuring crop safety [113,146].

Phytotoxic responses can vary depending on the type of essential oil and the plant species involved. For example, Ibáñez and Blázquez [147] found that tomato plants are more sensitive than cucumber to several commercial oils, with the radicle being most affected, an important detail when considering root-knot nematode control. Likewise, Alipour et al. [148] showed that encapsulating rosemary oil does not eliminate phytotoxicity but can alter its effects, including reducing chlorophyll content, increasing proline levels, and causing membrane damage.

By contrast, studies using solid plant amendments generally document the absence of phytotoxic effects; in some cases, they even report improvements in root growth. These findings indicate that the method of application of botanical products plays a critical role in their impact on host plants [95,103].

### 9.3. Effects on Non-Target Nematodes and Soil Microfauna

Botanical extracts not only affect plant parasitic nematodes. Many studies have reported their effects on free-living nematodes and other soil microfauna. For instance, some essential oils can reduce the populations of bacterivorous and fungivorous nematodes, which may in turn alter the structure and function of soil food webs [94,98,104].

Comparative studies indicate that these effects are not universal. Ntalli et al. [95] showed that essential oils decreased the abundance of non-target nematodes, whereas aqueous extracts and plant powders maintained or even increased bacterivorous nematode populations, suggesting a differentiated functional response. Similarly, Monokrousos et al. [103] reported that plant amendments reduced *M. incognita* populations without causing a collapse of free-living nematode communities, preserving taxonomic richness and favoring opportunistic groups associated with enriched soil systems [94].

### 9.4. Impact on Soil Microbiome and Mycorrhizal Associations

Applying plant extracts with nematicidal properties can also influence the soil microbiome and its functions. For example, Stamou et al. [102] found that using *Mentha spicata* essential oil changed the composition of microbial communities and reduced early colonization by arbuscular mycorrhizal fungi. It also affected the activity of enzymes involved in nitrogen cycling [94].

Additional research using lipid biomarkers and analyses of soil communities has demonstrated that certain plant amendments can increase bacterial biomass and promote less-stressed soil systems. Conversely, some botanical treatments have been observed to induce disturbances similar to those caused by synthetic nematicides [103,113]. These findings indicate that plant extracts are capable of reshaping soil microbial community structure and functional activity alike, with consequent implications for soil fertility and agroecosystem resilience. Nevertheless, knowledge remains limited regarding specific mycorrhizal taxa, the persistence of these effects, and their manifestation under field conditions.

### 9.5. Indirect Effects on Beneficial Insects and Aboveground Organisms

Most of the available evidence comes from studies on botanical insecticides rather than from nematicidal applications. However, there is consistent proof that essential oils and plant-based biopesticides can harm beneficial insects such as pollinators and natural enemies. For instance, researchers have found both lethal and sublethal effects in honey bees, including changes in behavior, movement, and detoxification-related enzyme activity [149,150].

Furthermore, recent research has revealed that some botanical insecticides are not highly selective towards natural enemies, especially during their larval stages. In some cases, phytotoxicity caused by these treatments can indirectly raise mortality rates in these beneficial organisms [151].

### 9.6. Methodological Biases in Ecotoxicological Evaluation

Assessing the ecotoxicological impacts of botanical nematicides poses significant methodological challenges. Most studies rely on short-term laboratory or in vitro tests, while long-term, functional, or field-based evaluations are much less common [94,113]. Some studies have found that results seen in the lab, such as nematicidal success, do not always translate to greenhouse or field trials, sometimes due to unforeseen phytotoxicity or ecological effects [146]. These limitations make it harder to generalize findings and underscore the importance of experimental approaches that account for multiple trophic levels and real-world soil functions.

### 9.7. Knowledge Gaps and Implications for Sustainable Use

Despite growing research, we still know little about the long-term effects of using plant extracts as nematicides, especially regarding the stability of soil communities, the health of mycorrhizal associations, and the extent to which these products are truly selective. Evidence so far suggests that compatibility with sustainable agriculture depends greatly on how these products are formulated, the dosages used, and the way they are applied. For this reason, it is essential to integrate ecotoxicological, phytotoxic, and functional assessments to ensure the responsible use of botanical extracts in the management of plant-parasitic nematodes.

## 10. Commercialization and Regulatory Challenges

The transition of botanical extracts with nematicidal properties from research laboratories to commercial markets presents significant challenges, which have constrained the diversity of available products. Although numerous plant species with biocontrol potential have been identified, only a limited number of active ingredients have reached the market. This limitation is primarily attributable to factors such as variability in plant chemistry, high production costs for sufficient raw material, and stringent international regulatory requirements.

As a result, the market for botanical bionematicides is highly concentrated (Table 3). Industry tends to favor products that are stable, consistently effective, and have a track record of meeting regulatory standards.

### 10.1. Major Botanical Active Ingredients in Commercial Products

A closer look at commercial product labels shows a clear trend: companies rely on certain groups of secondary metabolites with proven efficacy. One key example is azadirachtin, extracted from neem (*Azadirachta indica*), which is now a standard active ingredient for controlling plant-parasitic nematodes. Azadirachtin disrupts several metabolic processes in nematodes, mainly by interfering with chitin synthesis, protein production, and their overall physiological performance [110,154]. It is available in products with concentrations ranging from 0.15% (such as Achook) to 3–4.5% in high-purity formulations such as AzaGuard, Azatrin O, and EcoGarden.

Another important group is saponins, especially those from *Quillaja saponaria*, which are now common in technical applications. While commercial products often focus on total saponin content, research suggests that the best nematicidal results come from a synergy between saponins and other components in the extract [155]. These ingredients can be found in products like QL Agri 35 and Monterey Nematode Control, sometimes at concentrations as high as 30%.

Organosulfur compounds and phenols also play a major role because they are highly volatile and can spread easily through soil. Garlic extracts (*Allium sativum*) are commonly used due to their rich content of allyl sulfides, like diallyl disulfide and diallyl trisulfide, which are highly toxic to various plant-parasitic nematodes [156]. This approach has been standardized in products such as Nemguard SC. Additionally, the market now offers products containing phenylpropanoids and monoterpenes, including those found in cinnamon oil (*Cinnamomum verum*), as well as compounds such as thymol and geraniol.

### 10.2. Technical Barriers to Industrial Scale-Up

Scaling up and commercializing new botanical extracts is technically challenging, making them hard to compete with synthetic pesticides. One major issue is the natural chemical variability of these products. The phytochemical makeup of a plant can vary significantly depending on its genetics and environmental factors such as temperature, altitude, soil type, and sunlight. This variability makes it hard to produce uniform products that work the same way in every batch [157,158].

Beyond chemical variability, product stability presents another major challenge for commercialization. Many secondary metabolites degrade readily under sunlight (UV radiation) or upon temperature changes after application, so they do not persist long in the soil and have a shorter shelf life. This instability also drives up costs for storage, transport, and distribution [159]. The economic competitiveness of botanical nematicides relative to synthetic alternatives remains a critical barrier to adoption. Synthetic nematicides such as fluopyram and oxamyl benefit from decades of supply-chain optimization and high active-ingredient potency at low application rates, typically yielding a cost-per-hectare that remains difficult for botanical products to match. The primary cost drivers for botanical nematicides are threefold: (i) raw material supply variability, since wild-harvested or semi-cultivated plant sources introduce seasonal price fluctuations and quality inconsistencies; (ii) expensive quality control requirements to standardize chemically complex extracts across batches; and (iii) higher active-ingredient loads per application needed to achieve equivalent nematicidal suppression under field conditions [160,161]. However, botanical products become economically competitive under three conditions: high-value crops (e.g., strawberry, tomato, ornamentals) where market prices absorb higher input costs; integrated nematode management programs where botanical applications reduce the number of synthetic treatments needed, offsetting their individual cost premium; and regulatory environments that incentivize low-risk alternatives through faster registration pathways, reduced liability costs, or access to organic premium markets [162]. Advanced nanoformulations, by increasing biological activity per unit of active ingredient, offer the most direct route to closing the cost gap without sacrificing efficacy [120].

Another important issue is the need for a steady and sustainable supply of plant materials, which is often difficult to guarantee. Inconsistent or limited access to raw ingredients has slowed the market launch of many promising extracts that showed strong bioactivity in the lab [160].

### 10.3. Regulatory Frameworks and the Translational Gap

Besides technical issues, complicated regulatory systems are also a major barrier to the commercialization of new bionematicides. For example, in places like the European Union and the United States, registering a new plant-based extract can take two to four years because of strict rules about environmental risks, toxicity, and effects on non-target organisms [160,161]. The combination of regulatory hurdles and high costs has created a clear gap between research and real-world use. As a result, much academic work remains in the laboratory and does not progress to field validation or commercial products [93].

### 10.4. Strategies to Overcome Current Limitations

To overcome these challenges, new technological and regulatory approaches are needed. For instance, advanced formulations such as nanoencapsulation can shield active compounds from rapid environmental breakdown, improve their stability, and help them remain effective in the field [162]. Similarly, for botanical extracts to succeed commercially, it is important to use strict standardization protocols. These ensure consistent levels of active ingredients in each batch and are supported by reliable laboratory testing [93]. Finally, on the regulatory side, new categories, such as “basic substances” in the European Union, offer an opportunity to make it easier to register low-risk, naturally derived products. These changes can help bring eco-friendly alternatives into modern agriculture by simplifying the approval process [153].

## 11. Future Perspectives

Despite the extensive body of research on botanical nematicides, critical gaps remain in the field. While numerous plant species with documented nematicidal activity, active compounds with strong in vitro profiles, and mechanistic hypotheses have been reported, robust evidence demonstrating consistent translation of these findings into sustained, practical nematode control under agronomically relevant conditions is lacking. Future research should prioritize addressing this translational gap.

Mechanistically, the field needs multi-omics studies in the species that cause the most agricultural damage, principally the major *Meloidogyne* taxa that inflict economically significant losses across vegetable, fruit, and arable crop systems. Most of what is known at the molecular level comes from model organisms or from *B. xylophilus*, and extrapolating those results across taxonomically distinct plant-parasitic groups introduces uncertainty that currently cannot be quantified. Integrating transcriptomics, metabolomics, and proteomics with targeted biochemical validation would provide the kind of causal evidence from phytochemical exposure to specific molecular targets to measurable biological outcomes that the field presently lacks for most compounds. Hypotheses generated from docking should be viewed as preliminary leads that still require experimental validation, not findings in themselves; enzyme assays, receptor binding studies, or genetic interference approaches should follow docking predictions before those predictions are cited as mechanistic evidence.

Formulation deserves recognition as a primary research challenge, not a technical afterthought. The main factors limiting the performance of botanical nematicides in soil are already well recognized: volatility, chemical instability, poor water solubility, adsorption, and microbial breakdown. Technologies that can address these barriers, such as microencapsulation, nanoemulsification, and controlled-release matrices, have shown real promise in laboratory settings, but the critical evaluation work must happen at the scale of real soil, over complete crop cycles, at economically defensible application rates. It is also important to understand how different formulation choices influence non-target organisms and the environmental fate of these products, not just nematode suppression statistics.

The compositional complexity of botanical extracts is underexplored as a design opportunity. If synergistic interactions between extract components can be identified through systematic mixture-design experiments informed by chemical profiling and mechanistic knowledge, it may be possible to build formulations that achieve effective nematode suppression at lower total doses. This could help reduce phytotoxicity risk and limit exposure of non-target organisms, while potentially bringing some materials within a regulatory framework that crude high-dose extracts could not meet.

Ecological integration should become standard practice in efficacy evaluations. Suppressing a nematode population by a statistically significant margin tells you something, but it does not tell you whether the treatment maintained the soil community functions that support long-term crop productivity, whether it harmed free-living nematodes that contribute to nutrient cycling, or whether it disrupted mycorrhizal associations that affect plant nutrient uptake. Studies that evaluate multiple trophic levels together with functional indicators alongside population metrics provide a more defensible basis for recommending a product than nematode counts alone.

Finally, moving from discovery to practical deployment will require more deliberate consideration of regulatory requirements from early in the research process. In the EU and the US, registration timelines for novel biopesticide active ingredients can extend to four years, with requirements for extensive safety and environmental data. Research programs that operate without awareness of these demands until the point of potential commercial development waste both time and resources. Interdisciplinary teams combining chemistry, nematology, formulation science, ecotoxicology, and regulatory expertise are not a luxury but a practical necessity for any research aimed at producing products rather than papers.

## 12. Concluding Remarks

The evidence synthesized in this review answers the central question it set out to address: botanical extracts exert genuine, mechanistically diverse nematicidal activity against plant-parasitic nematodes through multiple simultaneous and interconnected pathways, but that mechanistic complexity, while scientifically distinctive, has not yet been successfully translated into reliable, sustained field performance. The critical implication is that the laboratory-to-field gap is not incidental; it is mechanistically explicable and therefore addressable through targeted research and formulation strategies.

At the molecular level, the nematicidal mechanisms identified across the reviewed literature are not independent events but form an integrated toxic cascade. Compounds that generate reactive oxygen species simultaneously overwhelm the nematode’s detoxification capacity glutathione S-transferases and ABC efflux transporters creating a positive feedback loop in which oxidative damage accumulates beyond the cell’s ability to neutralize it. Neuromuscular disruption through allosteric modulation of both acetylcholine receptors and GABA-gated channels produces rapid immobilization, which prolongs nematode contact with the phytochemical environment and allows upstream redox and metabolic disruptions sufficient time to become lethal. Vacuolar H^+^-ATPase inhibition compounds this scenario by disrupting the intracellular pH gradients required for vesicular xenobiotic efflux, further impairing the same detoxification pathways that oxidative stress has already compromised. The convergence of these upstream events on programmed cell death genetically confirmed through cep-1/p53, ced-3, and ced-4 loss-of-function studies in *Caenorhabditis elegans* provides the mechanistic endpoint that unifies the diverse toxic inputs. This interconnected architecture means a nematode population would need to simultaneously overcome multiple independent molecular mechanisms to develop resistance, a fundamentally harder evolutionary challenge than that imposed by conventional single-site nematicides, and one that constitutes a genuine and underexploited advantage of botanical nematicides as a class.

The same chemical reactivity that drives this multi-target toxicity, electrophilicity, membrane permeability, and redox activity is also mechanistically responsible for the instability of botanical compounds in soil. Volatility and chemical degradability are not unrelated limitations; they arise from the same molecular properties that make these compounds nematicidal. This mechanistic linkage reframes formulation not as a logistical afterthought but as a central scientific challenge: encapsulation and nanoemulsion technologies are justified precisely because they can preserve reactive chemical groups through the duration of the nematode life cycle without neutralizing their biological activity. A distinctive contribution of this review is the articulation, for the first time, of the causal linkage among chemotaxonomic plant family specialization, phytochemical reactivity, multi-target toxicity architecture, and soil stability limitations as a unified interpretive framework. The dominance of Lamiaceae, Asteraceae, Brassicaceae, and Meliaceae in the botanical nematicide literature reflects convergent biosynthetic evolution toward terpenoid and glucosinolate pathways that generate inherently multi-reactive molecules, a pattern that supports rational source selection and mechanism-guided formulation design. Realizing the field potential of botanical nematicides demands that future work prioritize multi-omics mechanistic validation in agronomically relevant *Meloidogyne* species, mechanism-preserving formulation science, and ecological integration as primary research endpoints. The translational gap is bridgeable but only when mechanistic understanding drives, rather than follows, product development.

## Figures and Tables

**Table 1:** Representative botanical extracts and metabolites discussed in this review, their source plants, target plant-parasitic nematodes, reported effects, and proposed modes of action.

Plant and Extract	Target PPN	Key Effect	Proposed Mechanism	Evidence Type	Ref.
*Artemisia nilagirica*, Essential oil	*M. incognita*	↑ Mortality	Oxidative stress; reactive oxygen species (ROS) accumulation → ROS-dependent programmed cell death (cep-1/p53, ced-3, ced-4)	Biochemical + genetic loss-of-function (*C. elegans*)	[63]
*Cinnamomum zeylanicum*/*Citrus aurantium*, Essential oils	*M. incognita*	J2 mortality; cholinergic impairment	Altered *Mi-ace-1*/*Mi-ace-2* expression; cholinergic neurotoxicity	Bioassay + gene expression profiling	[68]
*Juglans* spp. husks, 1,4-Naphthoquinone	*M. luci*	Mortality; ↓ egg hatching; ↓ reproduction	GABAergic disruption + detoxification suppression + ribosomal/spliceosomal transcriptomic collapse	Transcriptomics + prior bioassays	[64,69]
*Cinnamomum* spp. bark, Cinnamaldehyde	*M. incognita*	Swelling; turgor loss	vacuolar H^+^-ATPase (V-ATPase) inhibition → intracellular pH dysregulation; osmoregulatory failure	Phenotypic observation + scanning electron microscopy (SEM)	[70]
*Tulipa gesneriana*, Tulipalin A	*M. incognita*	Nematicidal activity	Covalent V-ATPase inhibition at conserved Cys residues; electrophilic, structure-dependent interaction	Structure–activity relationships (SAR) + biochemical assays	[71,72]
*Pinus* spp. resin, Rosin-based nematicide	*B. xylophilus*	↓ Viability; broad physiological damage	glutathione S-transferase (GST) inhibition + oxidative damage + lipid/carbohydrate metabolic disruption + apoptosis activation	Biochemical + multi-omics	[65]
*Piper longum*, Piperine	*B. xylophilus*	Curling; aggregation; death; ↓ fecundity	Putative glutamate-gated Cl^−^ channel (GluCl) interaction; paralysis phenotype resembling macrocyclic lactones	Phenotypic assays + molecular docking	[73]
*Zanthoxylum alatum*, Essential oil/nanoemulsion	*M. incognita*	↓ Infectivity; deterrence	Putative odorant-binding protein interference; disruption of chemosensory host-location	Bioassay + molecular docking	[74]

**Table 2:** Comparative efficacy of botanical and synthetic nematicides against plant-parasitic nematodes.

Nematode/Crop	Scale	Synthetic Nematicide	Botanical Result	Key Finding	Ref.
*M. incognita*/tomato	In vitro + greenhouse	Oxamyl LC_50_: 10.94 mg/L; ~99% suppression	Monoterpenes LC_50_: 36–73 mg/L	Botanical compounds highly active but less potent than oxamyl	[115]
*M. incognita*/tomato	In vitro + greenhouse	Abamectin EC_50_: 0.13 µg/mL	4-Quinolone alkaloids EC_50_: 0.09 µg/mL	Purified phytochemicals can exceed synthetic nematicide potency	[116]
*B. xylophilus*/pine	In vitro	Copper sulfate LC_50_: 0.07–0.15 mg/mL	Rosmarinic acid LC_50_: 0.95–1.18 mg/mL	Botanical compound active but ~10× less potent than chemical standard	[117]
*B. xylophilus*/pine	In vitro	Fenitrothion LC_50_: >10 mg/mL	Cinnamon essential oil (EO) LC_50_: 0.12–0.14 mg/mL	Essential oil far more potent than organophosphate (system-specific)	[118]
*M. incognita*/tomato	In vitro + greenhouse	Abamectin: 96.8% J2 mortality; ~75% pop. reduction	Botanicals: ≤89.8% J2 mortality; ≤28% pop. reduction	Synthetic nematicides more consistent; individual botanical treatments less effective	[108]
*M. incognita*/tomato	In vitro + greenhouse	Oxamyl: ~99% population reduction	Algal extracts (*Ulva fasciata*): ~75% reduction	Botanicals also induced plant defense genes; inferior in direct suppression	[119]
*M. incognita*/*lettuce and tomato*	Greenhouse	Fluopyram: −67% Pf (lettuce)	Azadirachtin: −71% Pf (lettuce)	Comparable in short-cycle crops; efficacy was not sustained in long-cycle tomato crops	[109,110]
*M. incognita*/tomato	Greenhouse	Velum Prime RF = 0.031	Integrated botanicals + bioagents RF = 0.035	Integrated botanical strategy nearly matched synthetic nematicide	[109]
*M. incognita*/tomato	Field	Fluopyram: Pf = 2.1	Nano-azadirachtin: Pf = 1.5	Nanoformulation matched and surpassed synthetic nematicide under field conditions	[120]
*M. incognita*/tomato	In vitro + microplot	Velum Prime: ~98% J2 mortality	Botanical + organic amendments + bioagents: comparable efficacy	Integrated approaches combining botanicals with biocontrol agents can match chemical control	[121]

**Table 3:** Commercially available botanical bionematicides. Concentrations are expressed as *w*/*w* unless otherwise noted. OMRI, Organic Materials Review Institute. Source: [151,152]. Commercially available plant extract-based bionematicides, including representative active ingredients, manufacturers, and countries of origin.

Commercial Name	Active Ingredients	Manufacturer	Country of Origin
Achook	Azadirachtin 0.1%	Organix Limited	Kenya
Almighty	Geraniol 4.5%	GroPro	United States
AzaGuard	Azadirachtin 3.0%	BioSafe Systems	United States
Azatrin O	Azadirachtin 4.5%	OHP Inc.	United States
Cedroz	Geraniol 12.1% and Thymol 4.1%	Eden Research plc	United Kingdom
Dazitol	Capsaicin 0.4% and Allyl isothiocyanate 3.7%	Champon Millenium Chemicals Inc.	United States
EcoGarden	Azadirachtin 1.2%	Parry America	United States
Eco-Nemguard	Garlic extract 45.0%	Organic Crop Protectants	Australia
Ecoworks EC	Cold-pressed neem oil 70.0%	ECOSTADT TECHNOLOGIES LLC	United States
Extracfin	Cinnamon oil 15.0%, neem 20.0%, epazote 25.0%, and yucca extract 10.0%	Formulabagro de México	Mexico
Kurandi	Epazote extract 30.0%, spearmint extract 20.0%, and rue extract 20.0%	Soluciones Sustentables en Agronegocios S.A. de C.V.	Mexico
Monterey Nematode Control	*Quillaja* saponins 8.6%	Lawn and Garden Products, Inc.	United States
Nemabiol Plus	Castor bean extract 5.1% and multibacterial complex	Fagro	Mexico
Nemacinux	Castor bean extract 40.0% and oregano extract 15.0%	Bioamin	Mexico
Nemagold	Marigold (*Tagetes* spp.) extract 80.0% and algae 10.0%	Atlántica	Spain
Nemakill	Cinnamon oil 32.0%, clove oil 8.0%, and thyme oil 15.0%	ExcelAg	United States
Nemax	Thymol 4.0% and Geraniol 12.0%	Brandt	Spain
Nemguard SC	Garlic extract 100.0%	Biogard	Italy
Nem-Over	Pine extract 25.0%, oregano extract 5.0%, and castor bean extract 30.0%	Comercializadora de Biorgánicos, S.A. de C.V.	Mexico
Promax	Thyme oil 3.5%	Bio Huma Netics Inc.	United States
QL Agri 35	*Quillaja* extract 30.0%	BASF Chile	Chile
Rutinal	Rue extract 100.0%	Safer Agrobiológicos	Colombia
Sterminar	Neem oil 10.0%, anise essential oil 10.0%, and cinnamon oil 8.5%	Fertilizantes e Insumos Agrícolas	Mexico

Sources: Compiled from the Organic Materials Review Institute (OMRI) Products List [153] and manufacturer technical product information.

## Data Availability

No new data were created or analyzed in this study. Data sharing is not applicable to this article.
